# Methane Adsorption and Transport in Tortuous Slit-like Nanochannels: A Molecular Simulation Study

**DOI:** 10.1021/acsomega.4c06969

**Published:** 2024-10-12

**Authors:** Jiang Wang, Jiaxuan Tang, Fuye Chen

**Affiliations:** College of Science, Guizhou Institute of Technology, Boshi Road, Dangwu Town, Gui’an New District, Guizhou 550025, China

## Abstract

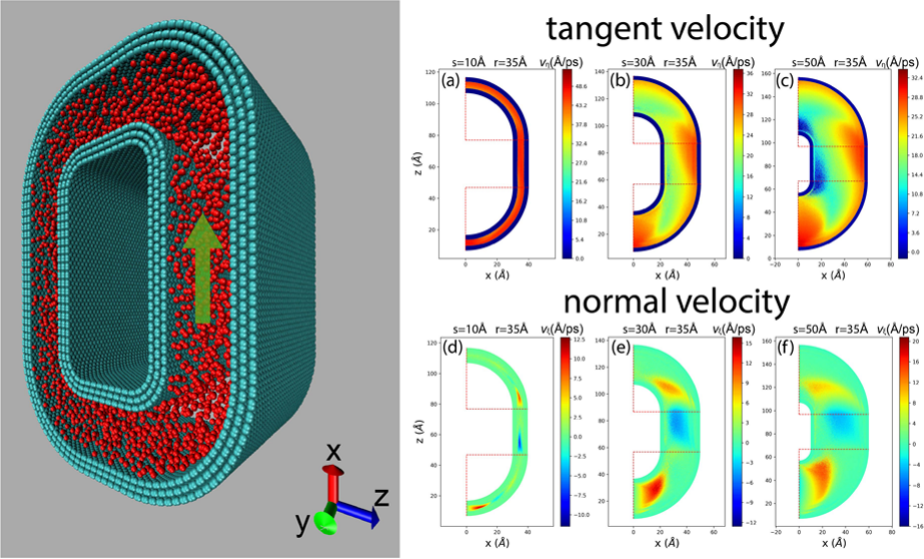

Tortuosity is a crucial characteristic of porous materials, such as the shale matrix where shale gas is stored. The presence of tortuous nanochannels significantly affects the adsorption and transport of nanoflows. In this research, we use molecular dynamics simulation (MD) to study the adsorption and transport properties of shale gas (methane) in a curved slit-like nanochannel constructed from bent graphene sheets. Our findings reveal that the curvature of the tortuous channel influences methane adsorption: convex surfaces exhibit stronger adsorption, while concave surfaces exhibit weaker adsorption; the discrepancy is amplified by the nanoflow. Additionally, nanoflow velocity is heterogeneously distributed within the curved channel, with higher tangential flow velocities observed near the entrance and the outer surface. We also identify a “bouncing effect”, where the nanoflow not only moves tangentially along the channel but also bounces between the inner and outer walls. Furthermore, methane in narrower channels exhibits higher tangent flow velocity and higher bouncing frequency but smaller flux, whereas larger curvature results in shorter channel length and smaller tortuosity, but the transport tangent velocity and flux are both reduced. The findings of this study can help in the better understanding of shale gas nanoflow properties in tortuous media and provide insights for simulating more general nonstraight nanoflows.

## 1 Introduction

As global energy consumption continues to rise, shale gas has emerged as a dominant energy resource due to its abundant reserves and relatively cleaner emissions compared to traditional fossil fuels.^1−9^ Primarily composed of methane, shale gas is predominantly stored in nanopores within the organic shale matrix, or kerogen.^4,10−14^ Advanced experimental methods have revealed that these nanopores form interconnected fractal networks, with sizes ranging from 1 to 100 nm and varying shapes such as triangles, circles, or slits.^15−23^ The realistic shale matrix is constituted of disordered and complex kerogen molecules, often containing multiple functional groups that impart roughness to the nanopores.^24,25^ The structure of these nanopores could been observed directly through experimental methods such as the scanning electron microscopy (SEM).^20,26,27^

A variety of experimental and numerical studies have revealed that nanopores and nanochannels within the shale matrix exhibit tortuous properties, with bends and curvatures throughout.^26,28−31^ Tortuosity, denoted as τ, is commonly estimated as τ = *C*/*L*, where *C* represents the length along the curved path within the nanochannels between two points, and *L* is the straight-line distance between those points.^32,33^ As with many other porous materials, tortuosity significantly affects the adsorption and transport of nanoflows in the media.^34,35^

Understanding the adsorption and transport properties of methane within nanopores and nanochannels is crucial for engineers involved in gas exploitation.^36−39^ Traditional experiments face challenges in exploring the complex behavior of methane in such confined spaces.^40−45^ Molecular dynamics (MD) simulation has proven to be an effective method for modeling and studying shale gas in nanopores.^41,42,44,46−57^ MD has been employed to investigate various aspects of nanopores affecting nanoflows, including the shape of the nanochannel’s cross-section,^58^ the roughness of pore inner walls,^59,60^ and the influence of functional groups^24,25^ and sticky layers^61^ on methane flow, and the enhancement of oil recovery using carbon dioxide.^62,63^ Despite these advancements, most previous studies have primarily focused on methane behavior in straight nanopores, leaving the behavior in more complex, tortuous structures less explored.

Several recent studies employing MD have explored the behavior of nanoflow in nonstraight nanopores. Zhang et al. simulated methane flow in a zigzag-shaped nanochannel and investigated the effects of the italic angle and turning angle, which define the structure of the nanochannel, on the flow properties. They find that italic angles enhance flow while smooth graphene surfaces exhibit higher velocities compared to rough surfaces. Regarding turning angles, they promoted methane flux in rough nanopores but hindered it in smooth nanopores.^64^ Ramírez et al. examined the effects of tortuosity and roughness in nanotubes, observing that both factors influence flow, with roughness playing a more prominent role. Moreover, larger curvatures and sudden changes in curvature were found to slow down the flow.^65^ In our recent work, the shale gas fluid in the curved single-walled carbon nanotube (SWCNT) is explored, and a flow turbulent vortex is observed at the entrance of the tortuous section.^66^

**Figure 1:**
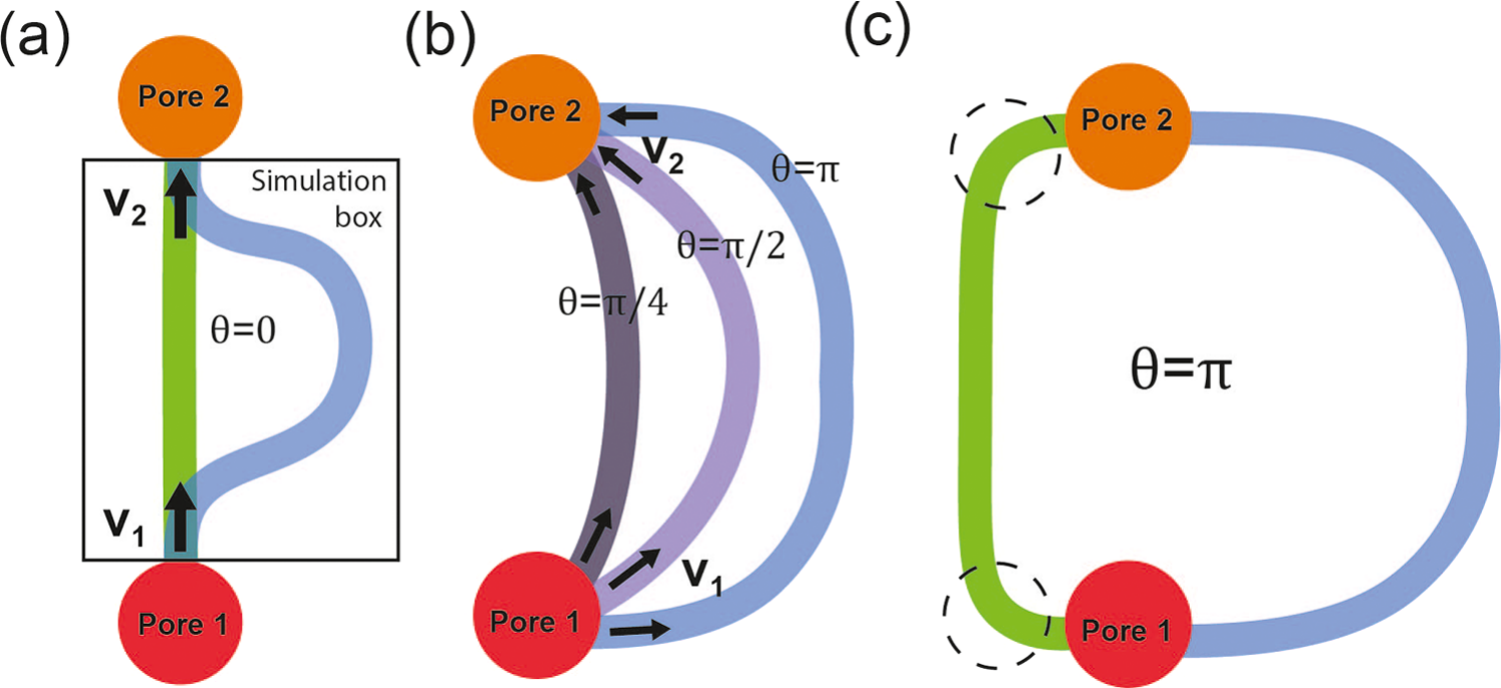
Overall description of the problem. Nanopore 1 and nanopore 2 are connected by distinct types of nanochannels. (a) Both channels exhibit identical entering (*v*_1_) and exiting (*v*_2_) velocities. The green channel features smaller tortuosity and curvature, whereas the blue channel displays larger tortuosity and curvature. Both can be simulated within a box with periodic boundary conditions. (b) In tortuous porous media, nanochannels linking two pores may possess different entering and exiting velocities, with the angle between *v*_1_ and *v*_2_ ranging from 0 to π. Such configurations cannot be simulated using a box with periodic boundary conditions. (c) Channels demonstrate differing entering and exiting velocities, with θ = π. The blue channel, characterized by larger tortuosity, is smoother and longer, while the green channel, with smaller tortuosity, is shorter and exhibits highly curved turnings as indicated by dashed circles.

To date, the majority of nanoflow simulations have been conducted within rectangular boxes employing periodic boundary conditions, as depicted in Figure 1a. In this configuration, the nanochannel entrance is periodically connected to the exit, resulting in identical flow velocities (both in direction and magnitude) at both ends. Consequently, the nanochannel is effectively considered infinitely long, accommodating both straight and nonstraight nanoflows.^47,50,67^ However, this assumption does not universally hold true, as demonstrated in Figure 1b. The angle θ between entering and exiting velocities may vary between 0 and π, and the velocities *v*_1_ and *v*_2_ are not necessarily equal. Consequently, rectangular periodic simulation boxes may prove inadequate in handling such scenarios.

In this study, our objective is to simulate shale gas nanoflow within a tortuous nanochannel. Instead of employing a regular periodic box, we propose a novel nanochannel structure by constructing a tortuous nanochannel using curved graphene sheets, forming circular periodic conditions. This approach enables us to simulate nanoflow within a curved nanochannel with varying entering and exiting flow velocities. Specifically, in our research, we set θ = π, as illustrated in Figure 1c. In this scenario, significant tortuosity corresponds to a longer, smoother path (depicted by the blue channel), while nanochannels with minimal tortuosity are shorter (shown by the green channel), typically characterized by highly curved turnings highlighted within dashed circles. This situation contrasts with θ = 0. Our investigation focuses on analyzing the impact of curvature on methane adsorption, examining the distribution of flow velocity within the curved nanochannel, and exploring how tortuosity and pore size influence the overall nanoflow flux. This research contributes to our understanding of nanoflow properties in tortuous porous materials such as shale gas within shale matrices, offering insights for simulating more generalized nonstraight nanoflows.

## 2 Methods

### 2.1 Nanochannel Structure

**Figure 2:**
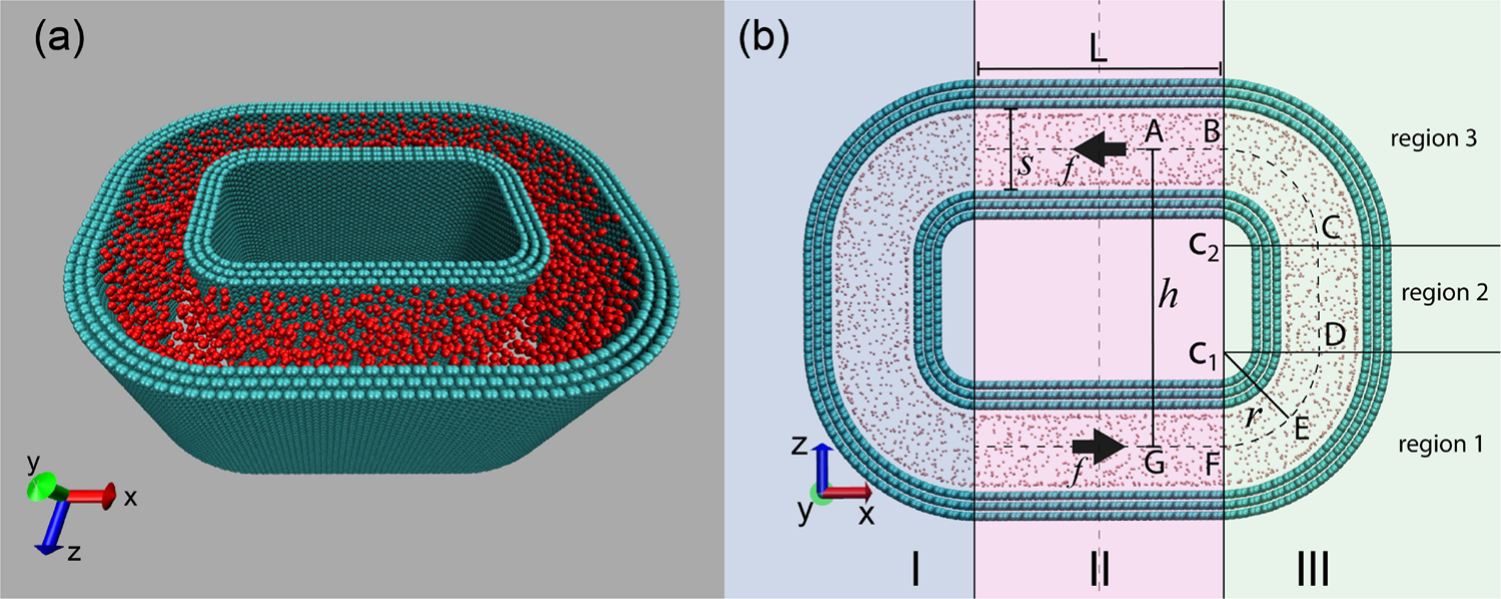
Sketch map showing the structure of the constructed nanochannel. (a) Snapshot of the nanochannel: carbon atoms are depicted in cyan, representing the graphene structure forming the walls of the nanochannel. Methane molecules are colored in red, indicating their presence within the nanochannel. (b) Structure parameters of the nanochannel: the nanochannel consists of three main sections: sections I and III are symmetrical to each other, while section II is straight and used for accelerating methane flow with a constant force *f* exerted on each methane molecule. The curved channel in section III comprises two circular regions (1 and 3), each spanning an angle of π/2, and a straight region 2. Methane flows into the nanochannel at the entrance (*F*) and exits through the exit (*B*), with the distance between the entrance and exit fixed at *BF* = *h* = 100 Å. The channel width is denoted as *s*, and the radius in regions 1 and 3 is represented as *r*. The length of region 2 is *CD* = *h* – 2*r*.

As illustrated in Figure 2a, the curved nanochannel is formed by walls consisting of three-layer graphene sheets. The height of the nanochannel in the *y*-direction is 40 Å, and the simulation box has a periodic boundary condition in the *y*-direction, indicating that this nanopore has a slit structure. The nanoslit is filled with methane molecules, depicted in red.

It is important to note that, although graphene has been extensively studied and proven to be an effective model for the nanochannel in shale matrix,^68^ the nanochannel we constructed is still far from replicating a realistic shale matrix. Real kerogen nanopores exhibit disordered complexity and functional groups, whereas our nanochannel is smooth and composed solely of graphene. The aim of this research is to investigate how a curved nanochannel impacts the adsorption and transport properties of shale gas. Therefore, we opted for a simple nanoslit structure to isolate the effects of curvature from other influencing factors.

The geometrical parameters of the nanochannel are illustrated in Figure 2b. The entire circular channel is symmetrical and consists of three sections: section II in the middle, and sections I and III on either side. Sections I and III are symmetrical to each other. Our analysis focuses solely on the curved Section III.

Section II is a straight channel with a length of *L* = 80 Å. During the simulation of methane flow, each methane molecule in the bottom channel is subjected to an external force of *f* in the direction of *x*, while molecules in the upper channel experience an opposite force of – *f* in the direction of – *x*. This setup induces a circular flow of methane molecules in a counterclockwise direction, with the angle between the entering velocity at point *F* and the exiting velocity at point *B* being π, as we only consider the curved channel in section III.

Section III consists of three distinct regions. Regions 1 and 3 are circular channels with centers at *C*_1_ and *C*_2_, respectively, each spanning an angle of π/2. The radius *r* is defined as the distance between the centers (*C*_1_ or *C*_2_) and the center of the slit: *r* = (*C*_2_*B* = *C*_2_*C* = *C*_1_*D* = *C*_1_*E* = *C*_1_*F*). The channel width remains constant at *s* along the entire loop. The distance between the entrance and exit of the curved channel is fixed at *h* = *AG* = *BF* = 100 Å, where *A*, *B*, *G*, and *F* are on the centerline of the channel. Region 2 is a straight segment with a length of *h* – 2*r*, which disappears if *r* = *h*/2.

In this research, we employed equilibrium and external field nonequilibrium molecular dynamics simulations (EF-NEMD)^47^ to investigate the impact of pore size (*s*) and curvature (1/*r*) on shale gas adsorption and flow properties. The pore sizes explored include *s* = 10, 30, 50 Å, and the radii examined include *r* = 15, 25, 35, 50 Å. Not all combinations of these *s* and *r* values are meaningful, as *r* must be greater than at least half of the channel width: *r* > *s*/2. Consequently, when *s* is large, small *r* values do not yield physically meaningful configurations. All possible *s* and *r* combinations are listed in Table 1.

**Table 1:** Simulation Parameters for Each Run in This Research^a^

	*s*	*r*	no. of	*f*
ID	(Å)	(Å)	methane	((kcal/mol)/Å)
1	10.0	15.0	842	–
2				0.05
3		25.0	900	–
4				0.0543
5		35.0	944	–
6				0.0594
7		50.0	1014	–
8				0.0669
9	30.0	25.0	2697	–
10				0.0543
11		35.0	2848	–
12				0.0594
13		50.0	3049	–
14				0.0669
15	50.0	35.0	4496	–
16				0.0594
17		50.0	4892	–
18				0.0669

a “–” in *f* column indicates that the external field is set to 0. Note that not all combinations of *s*, *r* values are meaningful, because *r* must be greater than at least half of the channel width: *r* > *s*/2, when *s* is large, small *r* will not render a physical meaningful configuration.

In this research, we model the nanochannels in kerogen using curved graphene based on several key considerations. First, the slit-like cross-sectional shape of our nanochannel is commonly observed in realistic kerogen nanochannels, as confirmed by experiments. Second, the attractive potential energy between graphene walls and methane molecules closely resembles that between actual kerogen and methane molecules, making graphene sheets a suitable analog.^68^ Third, graphene structures are easy to construct in simulations, and their structural parameters can be precisely controlled. This control is advantageous for quantitatively investigating the impact of channel geometry on methane behavior. Lastly, graphene is more accessible for experimental investigations. Realistic nanochannels in kerogen exhibit complex geometries with variable slit sizes and curvatures that are challenging to control, whereas the parameters of graphene sheets can be manipulated in experiments more easily.

By constructing the curved nanochannel as described above, we can simulate methane adsorption and transport within the curved channel without employing periodic boundary conditions, which allows the entering flow velocity to differ from the exiting flow velocity. This introduces a novelty in our work, distinguishing it from previous studies. However, it is worth noting that earlier works focused on simpler, straight nanochannels often incorporated more realistic kerogen models.

It is important to highlight that, although we use graphene sheets as a modeling framework for nanochannels within porous media like the shale matrix, our model is a simplified representation. In reality, nanochannels in shale matrices generally have rough surfaces and contain numerous disordered aromatic molecules and functional groups. Moreover, in other studies, the angle between the entering and exiting velocities can vary between 0 and π, while in our constructed nanochannel, this angle is fixed at π. These complexities add further intricacies to the fluid–solid interactions within the nanochannels.

Our research primarily aims to elucidate the effects of curved structures on methane nanofluid dynamics. To achieve this, we have deliberately chosen a simplified graphene model to isolate and investigate the impacts of curved geometry. By acknowledging the simplifications in our model, we can focus more precisely on the specific influence of nanochannel curvature on methane flow, drawing meaningful insights without the confounding effects of additional factors present in realistic shale matrix nanochannels. Once we have gained sufficient knowledge about the impact of curvature on methane transport, we can gradually introduce more complexities into the model. These complexities might include incorporating functional groups or surface roughness, altering the shape of the cross-section, or constructing curved nanochannels by compressing multiple individual kerogen molecules.^61,67,69^ This progressive approach will allow us to build a more comprehensive and accurate model of nanochannels in kerogen, thereby enhancing our understanding of methane behavior in these environments.

### 2.2 Molecular Dynamics Simulation

The molecular dynamics simulations were performed using the Large-scale Atomic/Molecular Massively Parallel Simulator (LAMMPS)^70,71^ under the canonical ensemble (NVT). The temperature was maintained at 400 K and controlled using a Nose-Hoover thermostat. To replicate the conditions of shale gas underground, the target pressure was set at 30 MPa, achieved by adjusting the number of methane molecules in each simulation: A trial number of methane molecules is initially inserted into the nanochannel for the simulation. The pressure is then computed using the built-in function in LAMMPS. If the measured average pressure is lower than the target pressure, the number of methane molecules is increased; otherwise, the number is decreased. After several iterations, the optimal number of methane molecules is determined when the system pressure converges to the target value. Since our simulations were conducted under the canonical (NVT) ensemble without pressure coupling, the actual pressures fluctuated around the target value, and the average pressures were close to the target with relative errors of less than 5%. Similar temperature and pressure were used by other researches on the shale gas property.^47,61,66,67^ The simulation integration time step was 2.0 fs, with production runs lasting 5.0 ns, which was sufficient for the system to reach equilibrium in adsorption simulations and for the flow to stabilize in EF-NEMD simulations. During the simulations, the atoms of the graphene sheets were kept frozen, allowing only the methane molecules to move.

There are two types of atoms in our simulations: C_Aro_, the aromatic carbon atom of graphene, and CH_4_, the united atom representing a methane molecule. The interaction between atoms of types *i* and *j* was determined using the Lennard–Jones potential function
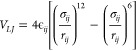
1with a cutoff of 1.2 nm. The values of ϵ_*ij*_ and σ_*ij*_ are listed in Table 2. For interactions between atoms of the same type, where *i* = *j*, we have ϵ_*ii*_ = ϵ_*i*_ and σ_*ii*_ = σ_*i*_.

**Table 2:** Parameters of the Lennard–Jones Functions for Each Atom Type Used in This Research

atom	ϵ (kcal/mol)	σ (Å)	mass (Da)
C_Aro_	0.07342	3.52053	12.0110
CH_4_	0.30187	3.70995	16.0430

For interactions between different atom types (*i* ≠ *j*), we used the geometrical rules to obtain the parameters:
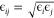
2
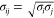
3

The parameters ϵ_*ij*_ and σ_*ij*_ are based on the Gromos54a7 force field,^72^ which is suitable for simulating hydrocarbons and biomolecules. Electrostatic interactions in our system were neglected since the graphene walls were kept frozen and both the graphene and methane united atoms were electro-neutral.

To verify the accuracy of the interaction parameters, we simulated methane gas in the bulk state at T = 400 K and P = 30 MPa, and compared the resulting density with experimental measurements. Our simulation yielded a density of 8.688 mol/L, while the experimental value is 8.793 mol/L,^73^ resulting in a relative error of 1.19%. This small error indicates that our model’s force field accurately captures the properties of methane under high-temperature and high-pressure shale conditions.

When simulating methane transport in the nanochannel, we utilize external field nonequilibrium molecular dynamics (EF-NEMD).^47,51,52,64^ In this method, an external force *f* exerted on each methane molecule in section II creates an effective pressure drop in the nanochannel. This approach has been widely employed in simulating nanofluid flow in nanochannels, primarily in straight channels.^47,51,52,56,64^

In straight channels, the entire system is accelerated, allowing the pressure difference between the two ends of the channel in the periodic simulation box to be rigorously obtained:^64,67^
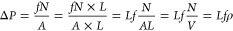
4where *f* is the force added to each molecule, *N* is the total number of molecules in the system, *A* is the area of the cross-section, and *L* is the length of the channel. The pressure gradient can be obtained as
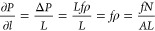
5

If only molecules in a part of the channel are subjected to external forces, and the length with external force is *L*, while the total length of the channel in the periodic box is *L*_0_, then the pressure difference in the channel remains the same as in eq 4, but *N* becomes the number of molecules with external force in the accelerated part *L*, rather than all molecules. *V* now represents the volume of the part of the channel with external forces. Based on eq 4, the pressure gradient can be obtained as
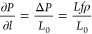
6

Note that in this case, *L* < *L*_0_, since *L* is only part of the whole channel.

In our system, the acceleration region comprises part of the entire channel, so we should refer to eq 6. The entire channel contains two periodically identical sections, each consisting of one acceleration part of length *L*, plus one curved half (Section I or III) whose length is π*r* + *h* – 2*r*. This implies that *L*_0_ = *L* + π*r* + *h* – 2*r*. Equation 6 holds true for both replications because they are periodically symmetrical to each other. Therefore, the pressure gradient in the entire channel is
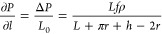
7

The pressure gradient ∂*P*/∂*l* generated by the external force *f* could drive methane fluid flow in the nanochannel in the counterclockwise direction. From eq 7, it is evident that both *f* and *r* can influence ∂*P*/∂*l*. In this research, we will set *f*_0_ = 0.05 (kcal/mol)/Å when *r* is minimal, specifically *r* = *r*_0_ = 15 Å. When *r* is larger, we ensure that ∂*P*/∂*l* remains constant across all situations. Thus, the force *f* for any other value of *r* can be determined by eq 9 as follows, and all corresponding forces *f* are listed in Table 1. Similar scheme for nanochannel construction and the derivation of the external force can be found in our recent work, for a nanochannel with a different cross-section shape.^66^
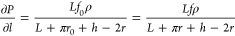
8
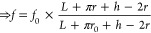
9

## 3 Results and Discussions

### 3.1 Effects of Curvatures on Methane Adsorption

**Figure 3:**
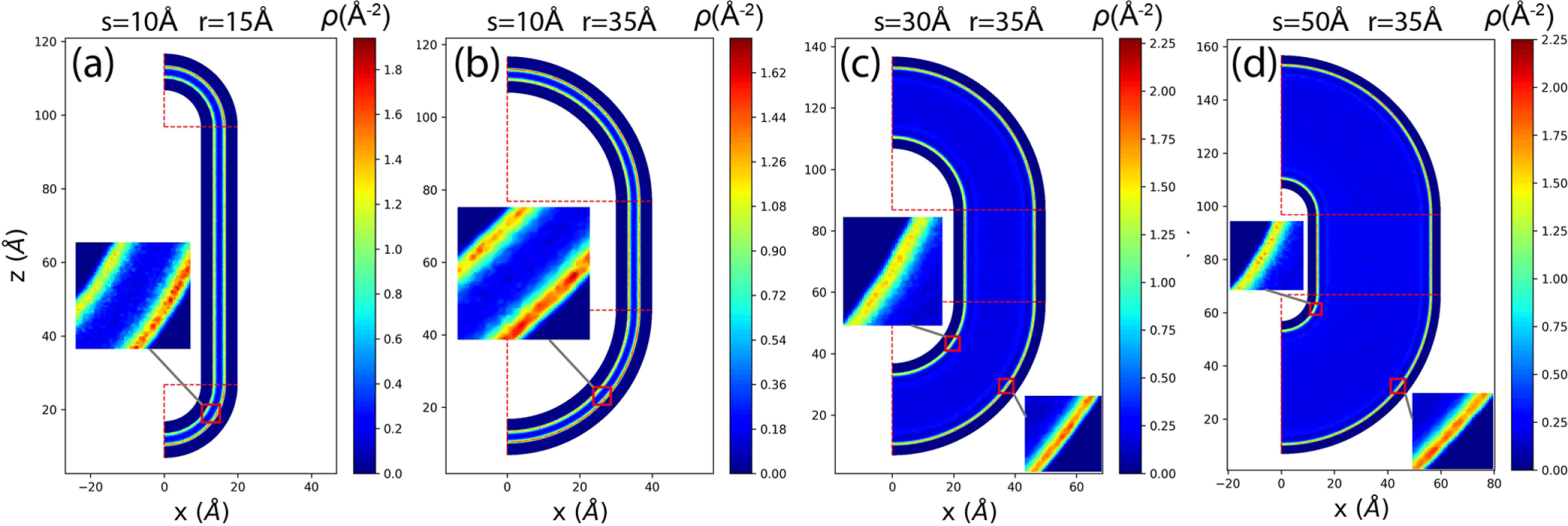
2D methane adsorption density in the (*x*, *z*) space for different typical cases: (a) (*s*, *r*) = (10 Å, 15 Å), (b) (*s*, *r*) = (10 Å, 35 Å), (c) (*s*, *r*) = (30 Å, 35 Å), (d) (*s*, *r*) = (50 Å, 35 Å). Panels (a) and (b) have the same *s* but different *r*, while panels (b), (c), and (d) have different *s* but the same *r*. Insets in each figure provide zoomed-in snapshots of typical regions. Horizontal and vertical red dashed lines indicate the radial boundaries of regions 1 and 3.

Methane molecules have a strong tendency to be adsorbed by the walls of nanopores, and this adsorption is influenced by the curvature of the walls. To investigate this effect, we calculate the 2D methane density in the *x* – *z* plane with the external force *f* turned off. The results for several typical combinations of (*s*, *r*) are presented in Figure 3. The unit for this 2D density is Å^–2^.

As shown in Figure 3a,b,c,d, high-density strips are consistently observed close to the walls along the nanochannel for different values of *r* and *s*. The outer walls have a larger radius, equal to *r* + *s*/2, while the inner walls have a smaller radius, equal to *r* – *s*/2. We observe that the density is higher near the outer wall. This observation becomes clearer when plotting the 1D density relative to the normal coordinates, i.e., along the radial coordinate *r*′ for regions 1 and 3, and along the *x* coordinate for region 2.

**Figure 4:**
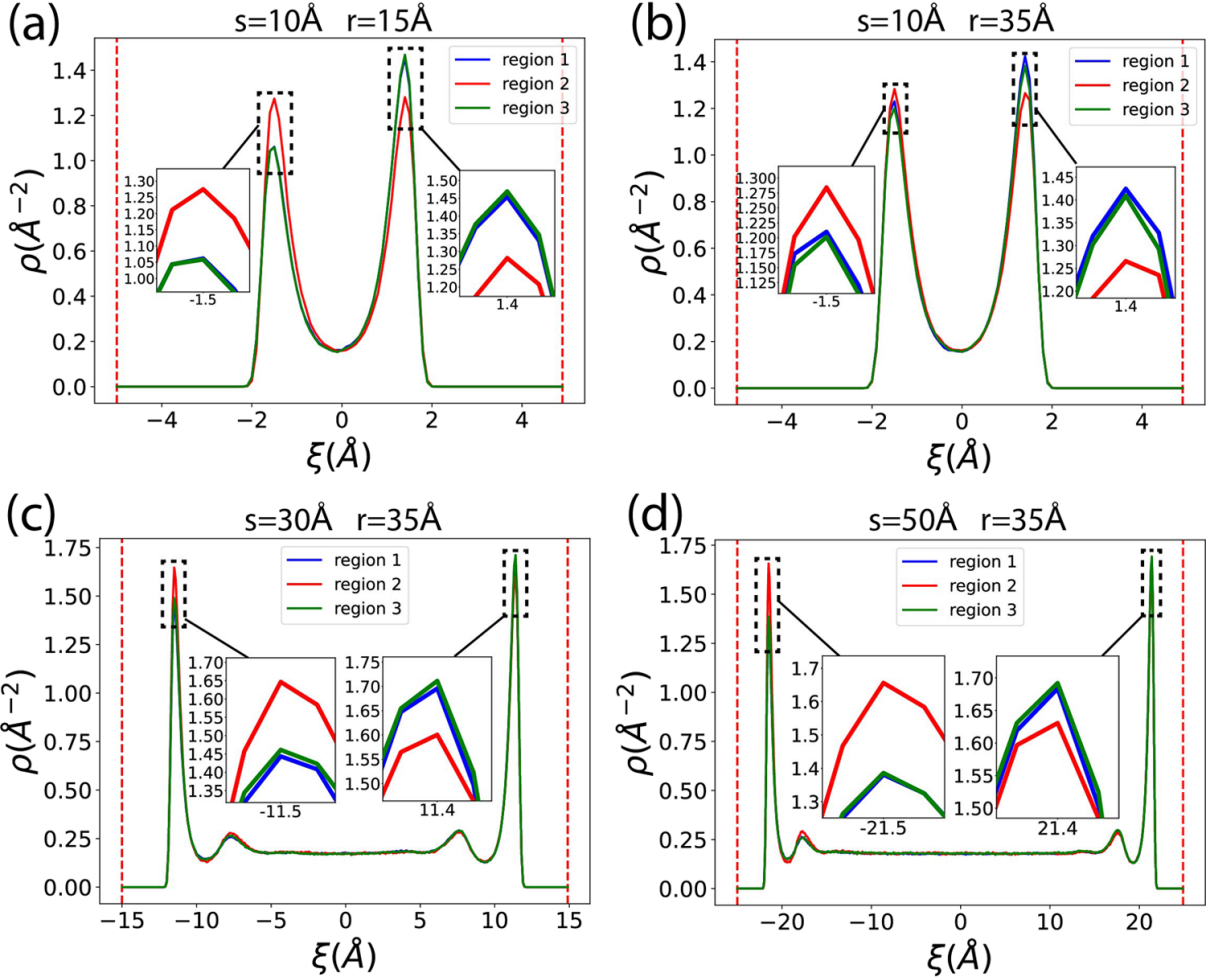
1D average methane adsorption density over the normal coordinate ξ. In regions 1 and 3, ξ represents the radial coordinate *r*′, and in region 2, ξ represents *x*. Panels (a) and (b) have the same *s* but different *r*, while panels (b), (c), and (d) have different *s* but the same *r*. Insets in each figure provide zoomed-in plots of curves that are too close to each other. Vertical red dashed lines indicate the surfaces of the nanoslit.

Figure 4 shows the relationship between the 1D averaged methane density and the normal coordinate ξ. In regions 1 and 3, the density is plotted along the radial direction, where ξ represents *r*′ (the radial coordinate, different from *r*, the radius of the channel). For region 2, the coordinate ξ stands for *x*. All ξ coordinates are shifted to be centered at 0, meaning the inner half of the channel has negative ξ values, and the outer half has positive ξ values. It is worthy to be clarified that the averaged 1D density ρ(ξ) is calculated numerically as
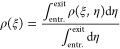
10which is the averaged number density displayed in the ξ space, obtained by taking the average of the 2D number density ρ(ξ, η) over the arc along the nanochannel from the entrance (entr.) to the exit, where η represents the tangent coordinate along the channel which is perpendicular to ξ, so the unit for ρ(ξ) is still Å^–2^.

We observe two peaks corresponding to two high-density layers close to the wall, with a gap of 0.0 density between the methane and the wall surface, which is denoted as vertical dotted lines. The width of this gap is about 3.0 Å, due to the repulsive interaction between carbon atoms in graphene and methane molecules at small distances. When the nanopore size *s* exceeds 30 Å, secondary peaks emerge.

In region 2, the inner peak (left side, with negative ξ close to the inner wall) and the outer peak (right side, with positive ξ close to the outer wall) have the same height, because region 2 is straight, both inner and outer walls are flat. Curves for region 1 and region 3 are almost identical and overlap to each other, this is because in the equilibrium simulation, region 1 and region 3 are symmetrical to each other, they exhibit identical adsorption properties to methane. However, in regions 1 and 3, the peaks near the inner layer are lower than those in region 2, and the outer layer has the highest peak. This indicates that methane molecules exhibit weaker adsorption on concave (inner) curved surfaces and stronger adsorption on convex (outer) curved surfaces, with flat surfaces showing intermediate adsorption strength. Similar phenomena have been observed in other studies.^74−78^

**Figure 5:**
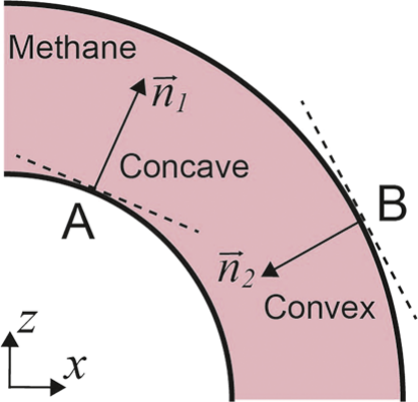
Definition of concave and convex surfaces: A concave surface is in a cap (∩) shape relative to the normal vector *n⃗*_1_ at point *A*, while a convex surface is in a cup (∪) shape relative to the normal vector *n⃗*_2_ at point *B*.

In this research, the concavity and convexity of the nanochannel surface are defined by referring to the 1D curve viewed in the 2D *x* – *z* space. The nanochannel has a cylindrical slit structure, so the Gaussian curvature of the surface is 0.0 everywhere.^79,80^ The curvature we defined is the inverse of the radius of the 1D arc in the 2D *x* – *z* space. As shown in Figure 5, the normal vectors at points *A* and *B* are perpendicular to the surface and point toward the methane inside the channel. At point *A*, the surface is curved downward into a ∩ shape relative to the normal vector *n⃗*_1_, making the surface at *A* concave. At point *B*, the surface is curved upward into a ∪ shape relative to the normal vector *n⃗*_2_, making *B* convex.

In Figure 4a,b,c,d, we observe that the discrepancy in adsorption between convex and concave surfaces varies with different curvatures (1/*r*) and nanochannel sizes *s*. We use *p*_*o*_/*p*_*i*_, the ratio between the outer peak height and the inner peak height, to represent the curvature effect on this adsorption discrepancy. A larger *p*_*o*_/*p*_*i*_ ratio indicates a more significant impact of curvature on adsorption, enhancing the discrepancy; in other words, a convex surface exhibits stronger adsorption than a concave surface.

**Figure 6:**
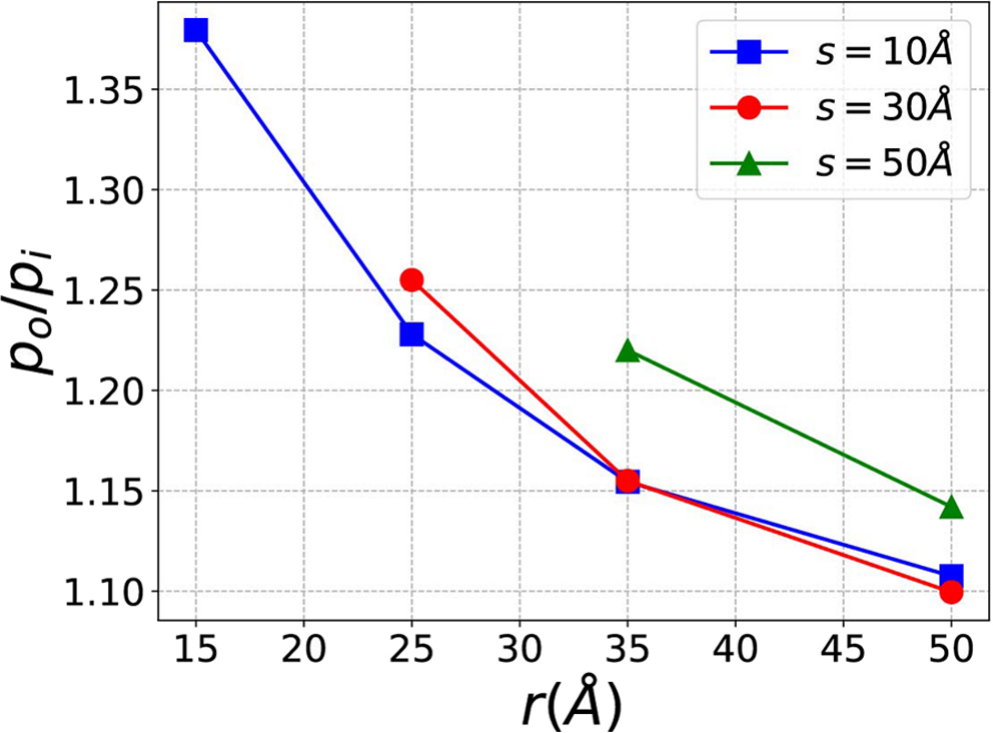
Ratios between the heights of outer adsorption peaks (*p*_*o*_) and inner adsorption peaks (*p*_*i*_) for different *r* and *s*. A larger *p*_*o*_/*p*_*i*_ indicates a stronger curvature effect on methane adsorption.

Figure 6 shows the *p*_*o*_/*p*_*i*_ values for all (*s*, *r*) combinations. The phenomenon is more prominent when *r* is small (*r* < 25 Å), where *p*_*o*_/*p*_*i*_ is large, indicating that larger curvatures lead to a more significant curvature effect on adsorption. As *r* increases, *p*_*o*_/*p*_*i*_ drops significantly, suggesting that larger *r* and smaller curvature result in weaker curvature effects on methane adsorption. When the nanopore size *s* is small (*s* ≤ 30 Å), *s* has a minimal impact, as indicated by the close proximity of the red and blue points. However, when the pore size *s* is large (*s* = 50 Å), the effect becomes more pronounced, as shown by the significant rise of the green points. This is because when *s* is large, the radius of the inner wall (*r* – *s*/2) can be quite small, while the radius of the outer wall (*r* + *s*/2) can be large and closer to a flat surface. This increases the difference between the inner and outer curvatures, resulting in a greater discrepancy in their adsorption abilities.

### 3.2 Impacts of Pore Sizes toward Flow Velocity

**Figure 7:**
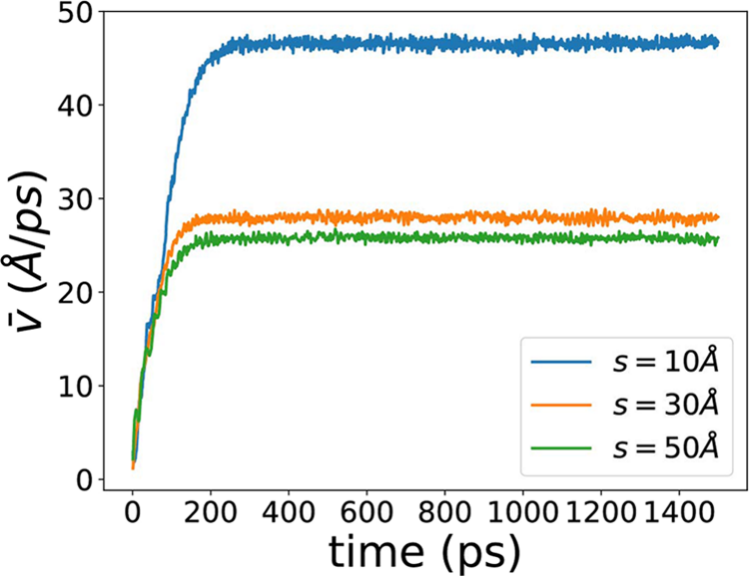
Average tangent velocity in section II as a function of time for different slit sizes with *r* = 35 Å.

When the external force is turned on in the EF-NEMD, methane molecules accelerate in the counterclockwise direction over time. We calculate the average methane flow velocity *v*_*x*_ in section II for the case of *r* = 35 Å and three different pore sizes. The data from the first 1500 ps of the 5000 ps full trajectory are displayed in Figure 7. It can be observed that all velocities initially increase from 0.0 Å/ps as time progresses. Eventually, they reach a plateau and stabilize at a constant value. This indicates that after an initial period of acceleration, the methane flow reaches a stable state, where the velocity remains unchanged. Furthermore, it is evident that narrower nanochannels exhibit higher final velocities and take longer to reach the plateau. Moreover, the observed velocities are significantly higher than the methane flow velocities in channels with roughness,^60,67^ this is because of the smooth feature of the nanoslit walls, which enhances the velocity of nano fluid, and this is observed by previous theoretical and experimental researches.^66,81−84^

**Figure 8:**
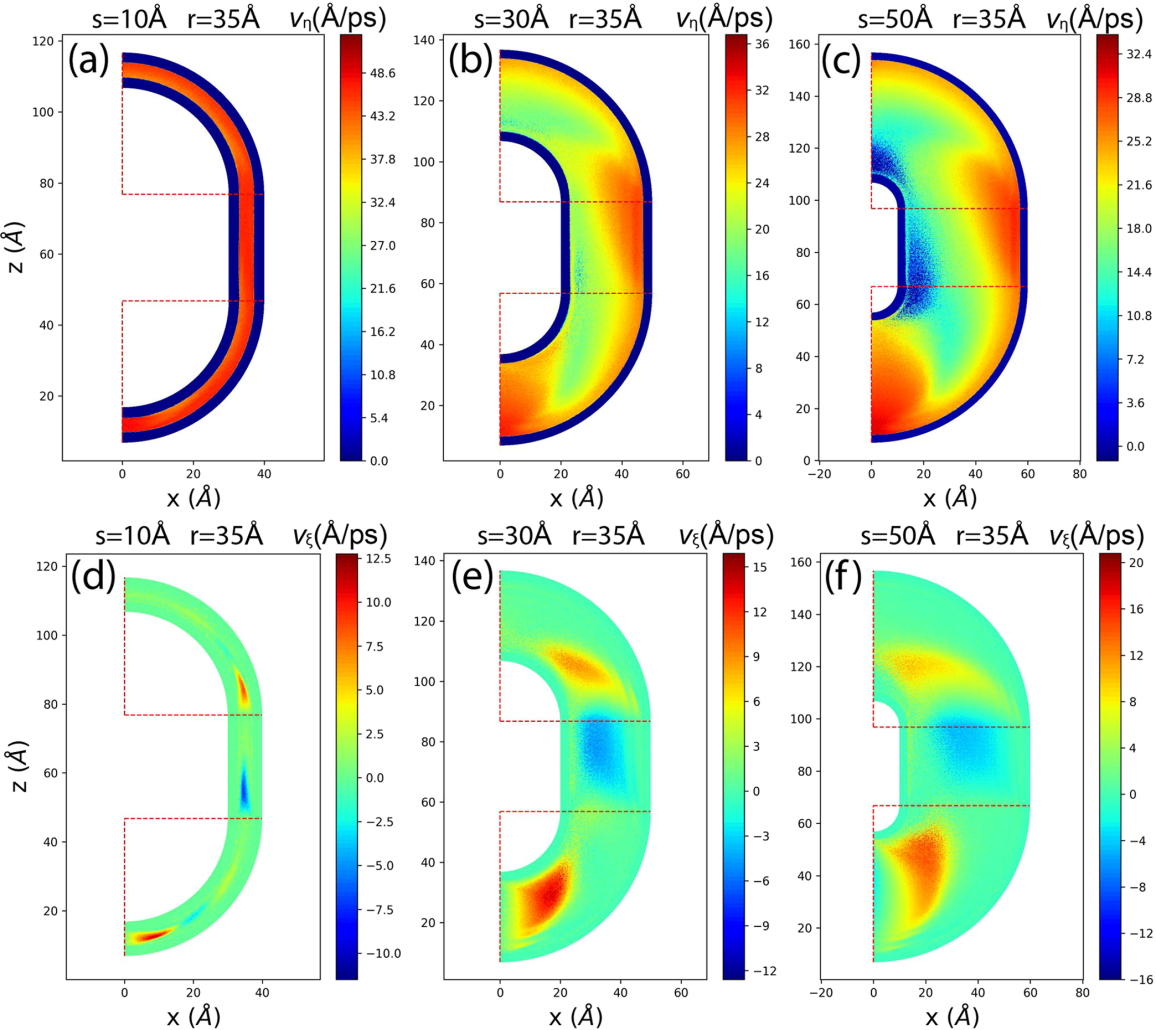
2D flow velocity distribution in the nanochannel with *r* = 35 Å and different *s*. (a)(b)(c) are for the tangent flow velocities *v*_η_. (d)(e)(f) are for the normal velocities *v*_ξ_. Horizontal and vertical red dashed lines are the radial boundaries of region 1 and 2.

The velocity of the flow can be decomposed into the tangent velocity *v*_η_ and the normal velocity *v*_ξ_, where η denotes the tangent coordinate along the channel and ξ is perpendicular to η. The tangent velocity is aligned with the path of the channel, while the normal velocity is perpendicular to the path. Figure 8 depicts the distribution of tangent and normal velocities for channels with *r* = 35 Å and different *s*.

In Figure 8a,b,c, we observe that when the channel is narrow, the tangent velocity *v*_η_ is quite uniformly distributed, and the velocity is the highest. As *s* increases, the tangent velocity distribution becomes less uniform. Velocities of methane near the entrance and the outer wall are higher compared to those near the inner walls and the exit. This indicates that the overall tangent velocity decreases after methane traverses the entire curved channel, with methane molecules moving faster near the outer wall than the inner wall.

Figure 8d,e,f show the normal velocity *v*_ξ_ when *r* = 35 Å. The normal velocity oscillates between positive and negative values along the channel, indicating that methane flow not only moves along the channel but also bounces back and forth between the inner and outer walls. As shown in Figure 8e,f, the *v*_ξ_ profile changes with increasing *s*, resulting in a longer tangent distance for one bounce period compared to when *s* is small. This implies that narrower channels have shorter bouncing wavelengths and higher frequencies. Additionally, peak values of *v*_ξ_ increase with larger *s*, indicating that methane flow in narrower channels has smaller normal velocities, allowing the flow to be more “targeted” toward the tangent direction, leading to higher tangent velocities. Similar behaviors are observed for cases where *r* = 15, 25, and 50 Å, as shown in Figures S1 and S2 in the Supporting Information. Notably, this “bouncing effect” is not observed in studies of nanoflow in straight channels.

We notice that the peak transport velocity of methane exceeds 10 Å/ps, which is significantly higher than the methane flow velocity observed in nanochannels with roughness.^41,42,44,46−57,64,65^ The smooth graphene walls of the nanochannel significantly reduce friction, enhancing flow velocity and flux. This phenomenon has been widely observed in previous research. In our earlier work, we demonstrated that methane flow velocity in straight nanochannels is significantly higher with smooth graphene walls compared to rough walls.^60^ Other experimental and theoretical studies also show that single-walled carbon nanotubes (SWCNTs), which are smooth and similar to graphene sheets, can enhance flow velocities up to 10^3^ m/s.^81−84^

### 3.3 Impacts of Curvature toward Flow Velocity and Flux

**Figure 9:**
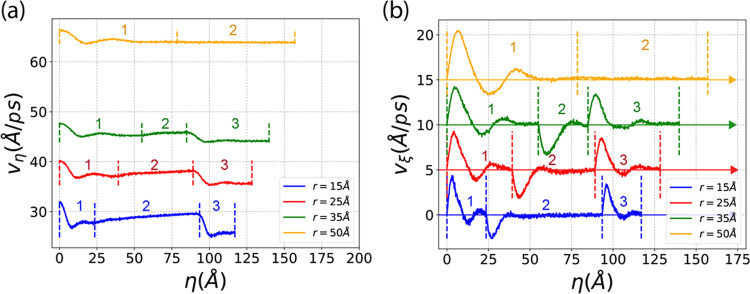
Tangent and normal flow velocities for nanochannels with *s* = 10 Å and different *r*. (a) Tangent flow velocity along the path of the nanochannel η, vertical colored dashed lines indicate boundaries of each region. (b) Normal flow velocity along the path of the nanochannel η. From *r* = 15 Å to *r* = 50 Å, each curve is lifted up by 5 Å/ps consecutively after the previous one to avoid the overlapping.

The curvature (1/*r*) significantly impacts flow velocity and flux. To illustrate this, we calculated the averaged tangent velocity (*v*_η_) and normal velocity (*v*_ξ_) for channels with *s* = 10 Å and various *r* values, plotting them along the channel coordinate η. As shown in Figure 9, the channel coordinate η is divided into three regions: region 1, region 2, and region 3, located on the left, center, and right sides, respectively, and separated by vertical dashed lines. Notably, when *r* = 50 Å, region 2 disappears because the length of the straight region becomes zero (*h* – 2*r* = 0 when *r* = *h*/2 = 50 Å).

Figure 9a shows the tangent velocity (*v*_η_) along the channel coordinate (η). We observe that smaller curvature (larger *r*) results in higher tangent velocity. There is a noticeable velocity drop at the beginning of regions 1 and 3, attributed to the sudden change in curvature, with smaller *r* values causing larger drops. Upon entering region 2, there is no velocity drop, and the velocity increases gradually in this straight section. This indicates that when straight flow enters a curved channel, a hindrance reduces the velocity, whereas curved flow entering a straight channel experiences no such impeding effect. Additionally, despite the overall channel length for *r* = 50 Å being the longest, its velocity is the highest. Conversely, the shortest channel (*r* = 15 Å) has the lowest velocity. This implies that highly curved turnings in the short channel significantly impede flow, thus reducing the transport velocity.

Figure 9b shows the normal velocity (*v*_ξ_) along the channel coordinate (η). Initially, all four curves overlap and start from 0.0 Å/ps. To improve clarity and avoid overlap, each curve is shifted upward by 5.0 Å/*ps* relative to the previous one. The normal velocity oscillates between positive and negative values along the channel due to the bouncing effect, with strong stimulation at the entrance of each region caused by the sudden change in curvature. When straight flow enters a curved channel, a positive *v*_ξ_ pulse is generated, whereas a negative *v*_ξ_ pulse is created when curved flow enters a straight channel. For the case of *r* = 50 Å, there is no straight region, so the curvature remains constant along the entire channel, resulting in no *v*_ξ_ stimulation except for the initial one at the entrance of region 1.

**Figure 10:**
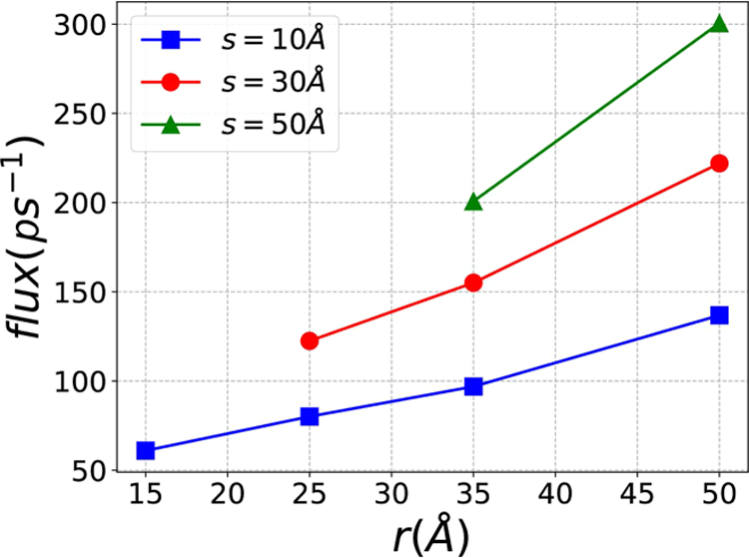
Flux of the flow in the nanochannel with different *s* and *r*.

We also calculate the flux of the flow for all values of *r* and *s*. The flux is defined as the number of methane molecules that flow across the cross-section of the channel per picosecond. As shown in Figure 10, larger pore sizes (*s*) result in larger fluxes. Additionally, for all pore sizes, as curvature decreases (i.e., *r* increases), the flux also increases. The tortuosity of the curved channel can be estimated as τ = *C*/*h*,^32,33^ where *C* is the arc length along the channel, and *h* is the distance between the entrance and the exit of the channel. In our case:
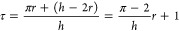
11

As *r* increases, the overall channel length also increases, resulting in greater tortuosity. This suggests that channels with high tortuosity may exhibit larger flux, a counterintuitive finding. Intuitively, one might expect greater flow impedance in a longer channel, leading to smaller flow flux. However, our results indicate that if velocities are opposite at the entrance and exit, longer channels may have smaller flow impedance and larger fluxes. This is because shorter paths with lower tortuosity are likely to contain highly curved, sudden turnings, which significantly impede the overall flux by reducing the flow velocity.

In summary, our findings suggest that while high tortuosity generally implies longer channels and thus intuitively greater resistance, in practice, smoother curves and fewer abrupt changes in direction can facilitate greater flow efficiency, resulting in higher fluxes despite the increased path length. This highlights the importance of considering both the geometric complexity and the specific nature of the flow dynamics within nanochannels.

### 3.4 Effects of Flow toward Adsorption

**Figure 11:**
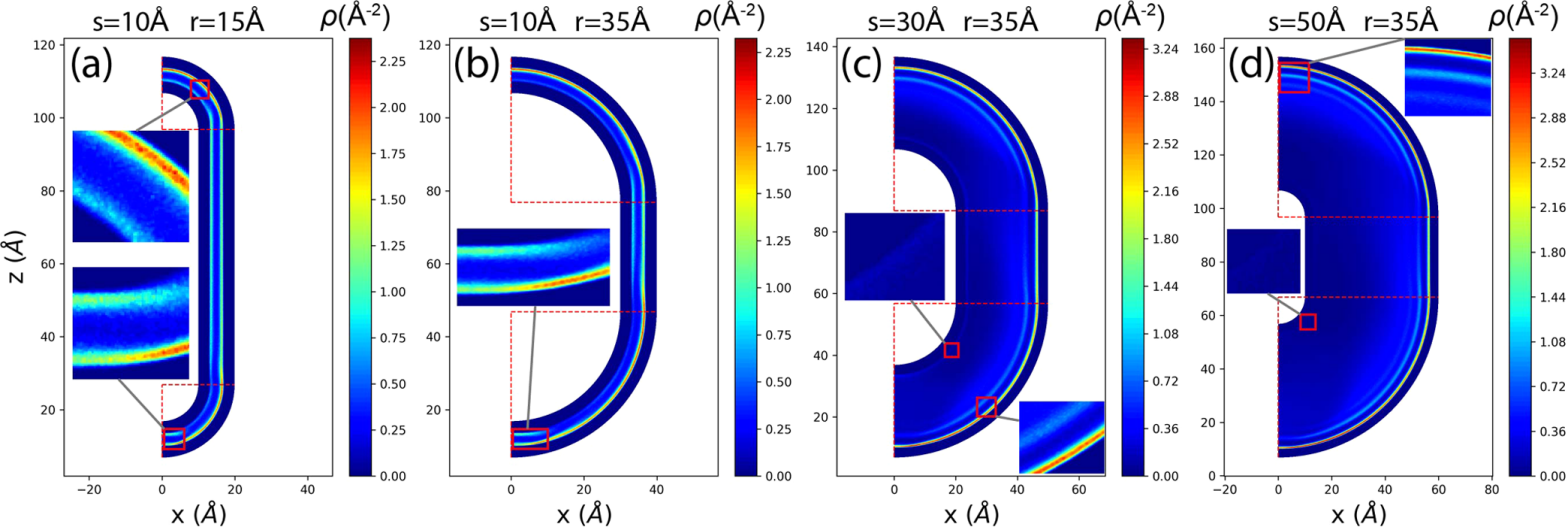
When the flow is turned on, 2D methane adsorption density in the (*x*, *z*) space for different typical cases: (a) (*s*, *r*) = (10 Å, 15 Å), (b) (*s*, *r*) = (10 Å, 35 Å), (c) (*s*, *r*) = (30 Å, 35 Å), (d) (*s*, *r*) = (50 Å, 35 Å). Panels (a) and (b) have the same *s* but different *r*, while panels (b), (c), and (d) have different *s* but the same *r*. Insets in each figure provide zoomed-in snapshots of typical regions. Horizontal and vertical red dashed lines indicate the radial boundaries of regions 1 and 3.

When the nanoflow is turned on, the adsorption properties of methane are altered. Figure 11 shows the 2D methane density in the *x* – *z* plane for some typical combinations of (*s*, *r*) when the force *f* is applied. In the bottom region of Figure 11a,b, a horizontal high-density region appears at the entrance of the nanochannel, caused by the inertial motion of the flow in the *x* direction upon entering the curved channel. From Figure 11c,d, we observe that the high-density strip near the inner wall almost disappears, and the density near the outer wall is enhanced. The density at the exit (region 3) is also higher than at the entrance (region 1), and a third adsorption peak can be observed near the outer wall of the exit, as shown in the inset of Figure 11d.

**Figure 12:**
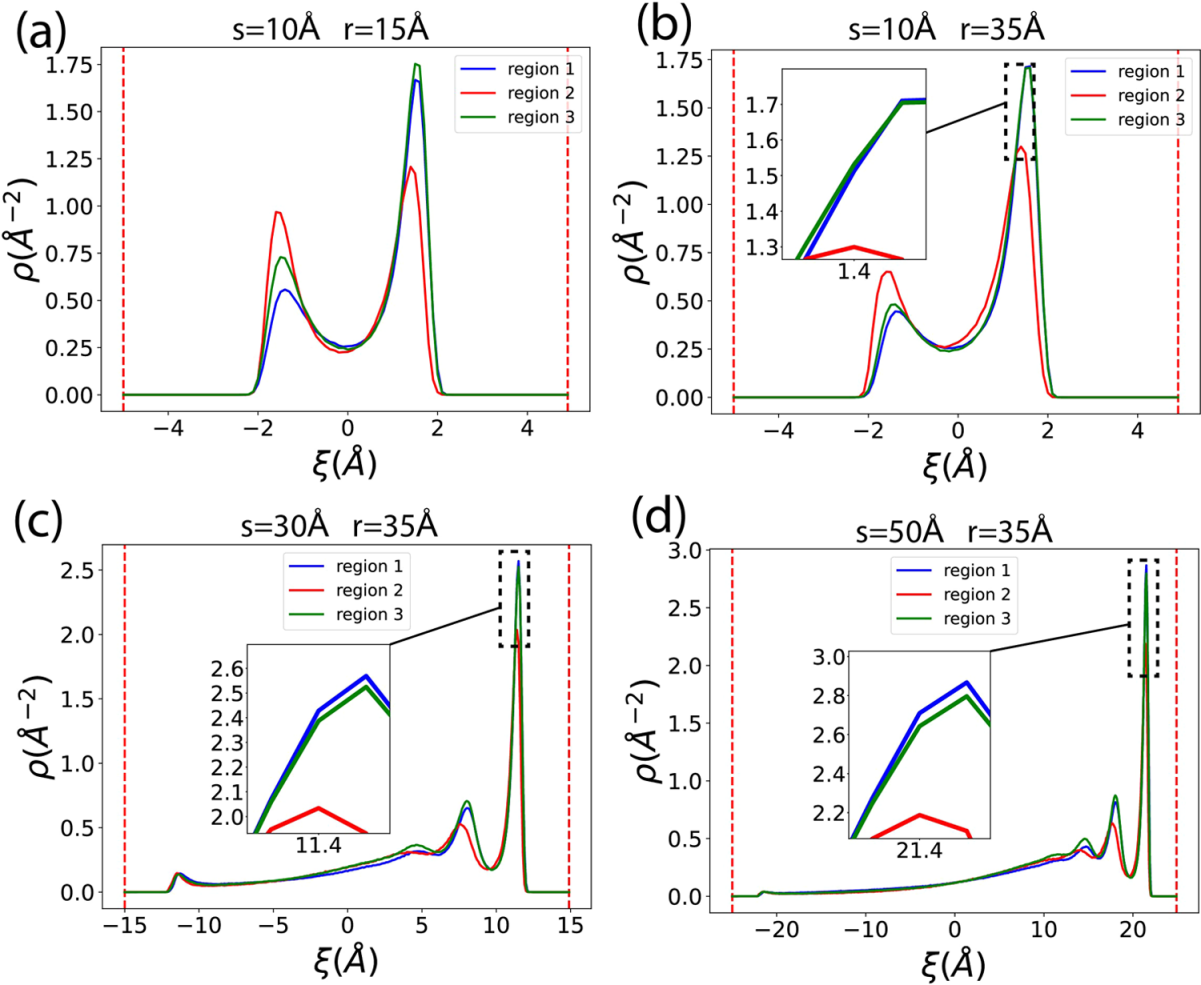
1D average methane adsorption density over the normal coordinate ξ when the flow is turned on. In regions 1 and 3, ξ represents the radial coordinate *r*′, and in region 2, ξ represents *x*. Panels (a) and (b) have the same *s* but different *r*, while panels (b), (c), and (d) have different *s* but the same *r*. Insets in each figure provide zoomed-in plots of curves that are too close to each other. Vertical red dashed lines indicate the surfaces of the nanoslit.

Figure 12 illustrates the relationships between the averaged adsorption density and the normal coordinate ξ in each region for different *r* and *s*. Similar to Figure 4, ξ represents *r*′ in regions 1 and 3, and *x* in region 2. All ξ coordinates are shifted to be centered at 0. We observe that for all cases, compared to Figure 4, the inner peaks (with negative ξ) are lowered, and the outer peaks (positive ξ) are enhanced. Even in the flat, straight region 2, the two peaks are no longer identical. In Figure 12a,b, when *s* = 10 Å, the curves in the entrance part (region 1) and the exit (region 3) also deviate from each other. In Figure 12c,d, when the nanochannel is large, third peaks emerge near the outer wall, while peaks near the inner wall almost disappear.

All these phenomena are due to the impact of the flow. The flow’s inertia causes it to move toward the farther part in a curved channel, increasing the adsorption density near the outer walls while creating a low-density region near the inner walls.

## 4 Conclusions

In this research, we studied the adsorption and transport behavior of methane fluid in a tortuous nanochannel using molecular dynamics simulations. A number of calculations and analyses were conducted, leading to the following conclusions:

Methane adsorption onto graphene depends on the surface curvature: convex surfaces exhibit the strongest adsorption, concave surfaces the weakest, and flat surfaces have intermediate adsorption ability.

The adsorption of methane onto curved graphene is influenced by the flow: adsorption near the outer walls is enhanced, while adsorption near the inner walls of the channel is weakened.

Methane flow velocity is heterogeneously distributed in the curved channel: methane near the outer surface and the entrance exhibits high tangent velocities, whereas it shows lower tangent velocities near the inner wall and the exit. The bouncing effect is observed, with the normal velocity oscillating between positive and negative values along the channel.

For nanochannels with the same curvature (1/*r*), methane in narrower channels exhibits higher tangent flow velocity and higher bouncing frequency, but smaller flux. For the same channel size, larger curvature (small *r*) results in shorter channel length and smaller tortuosity, but the transport tangent velocity and flux are both reduced because highly curved turnings hinder the flow and decrease the transport velocity.

This research is helpful for understanding the properties of shale gas in shale matrix with tortuosity and provides insights for simulating other nonstraight nanoflows. Future work could focus on channels with different cross-sectional shapes, such as circular or triangular, and on adding roughness and functional groups to the wall surface, making the channel more closely resemble realistic nanochannels found in shale matrices.

## Supporting Information Available

2D flow velocity distribution in the nanochannel with other *s* and *r* values (PDF)
