# Supplementary material for: Methane Adsorption and Transport in Tortuous Slit-like Nanochannels: A Molecular Simulation Study
Source: ACS Omega. 2024 Oct 12;9(42):43093–105. doi: 10.1021/acsomega.4c06969 (PMC11500140; doi:10.1021/acsomega.4c06969)
Supplement: Supplementary file 1 — ao4c06969_si_001.pdf [file ao4c06969_si_001.pdf]

SUPPORTING INFORMATION
Methane Adsorption and Transport in Tortuous Slit-Like Nanochannels: A Molecular Simulation Study

Jiang Wang,* Jiaxuan Tang, and Fuye Chen

College of Science, Guizhou Institute of Technology, Boshi Road, Dangwu Town, Gui'an New District, Guizhou, 550025, China

E-mail: cwangjiang@git.edu.cn

SUPPORTING FIGURES

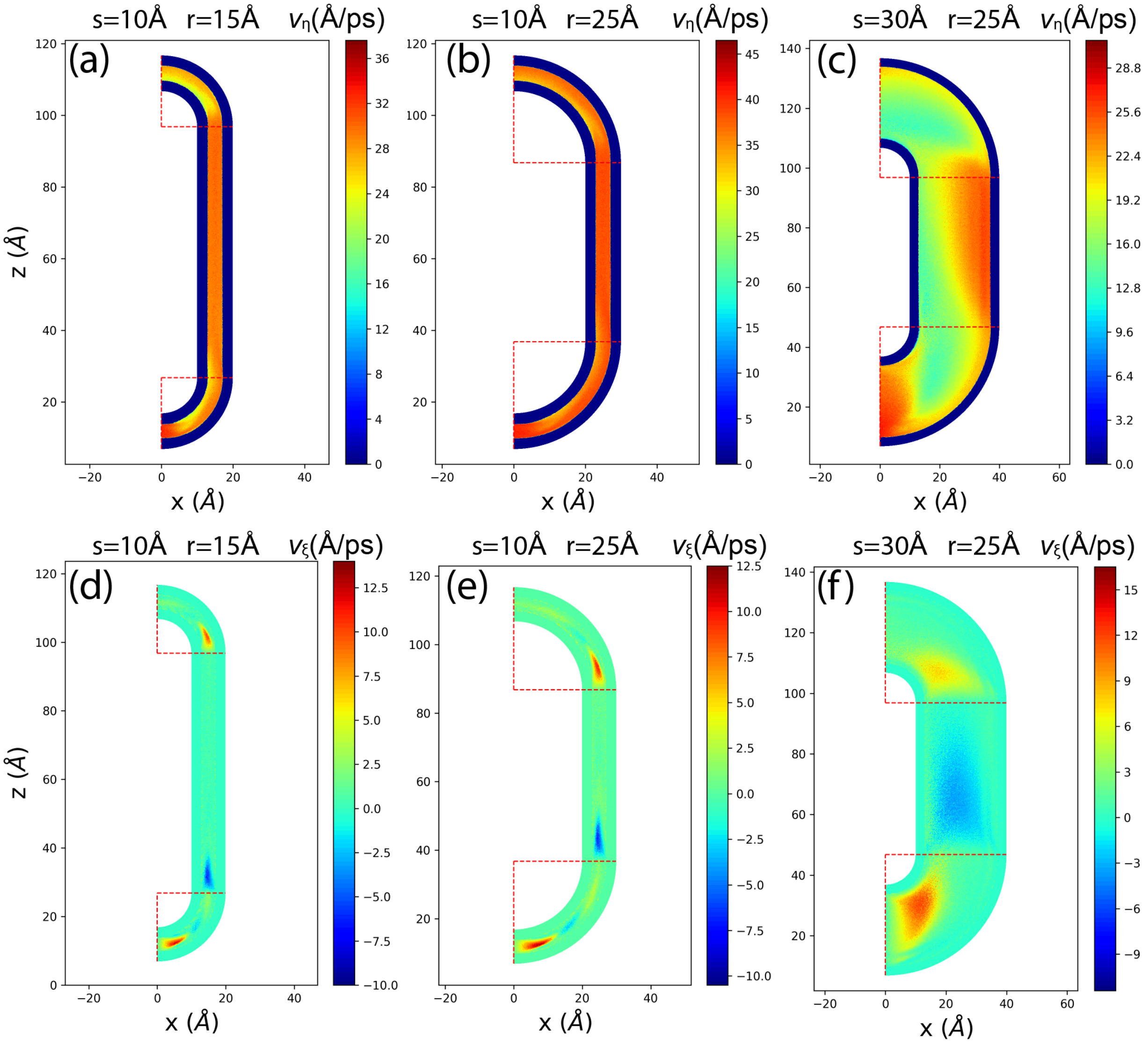

Figure S1: 2D flow velocity distribution in the nanochannel with different s, r values. (a)(b)(c) are for the tangent flow velocities. (d)(e)(f) are for the normal velocities. (a) and (d) are for $r = 50$Å, in this case, as shown in Table. 1 in the main text, s can only be 10Å. (b)(c)(e)(f) are for the case of $r = 25$Å, and (b)(e) are for $s = 10$Å, and (c)(f) for $s = 30$Å. Horizontal and vertical red dashed lines are the radial boundaries of region 1 and 3.

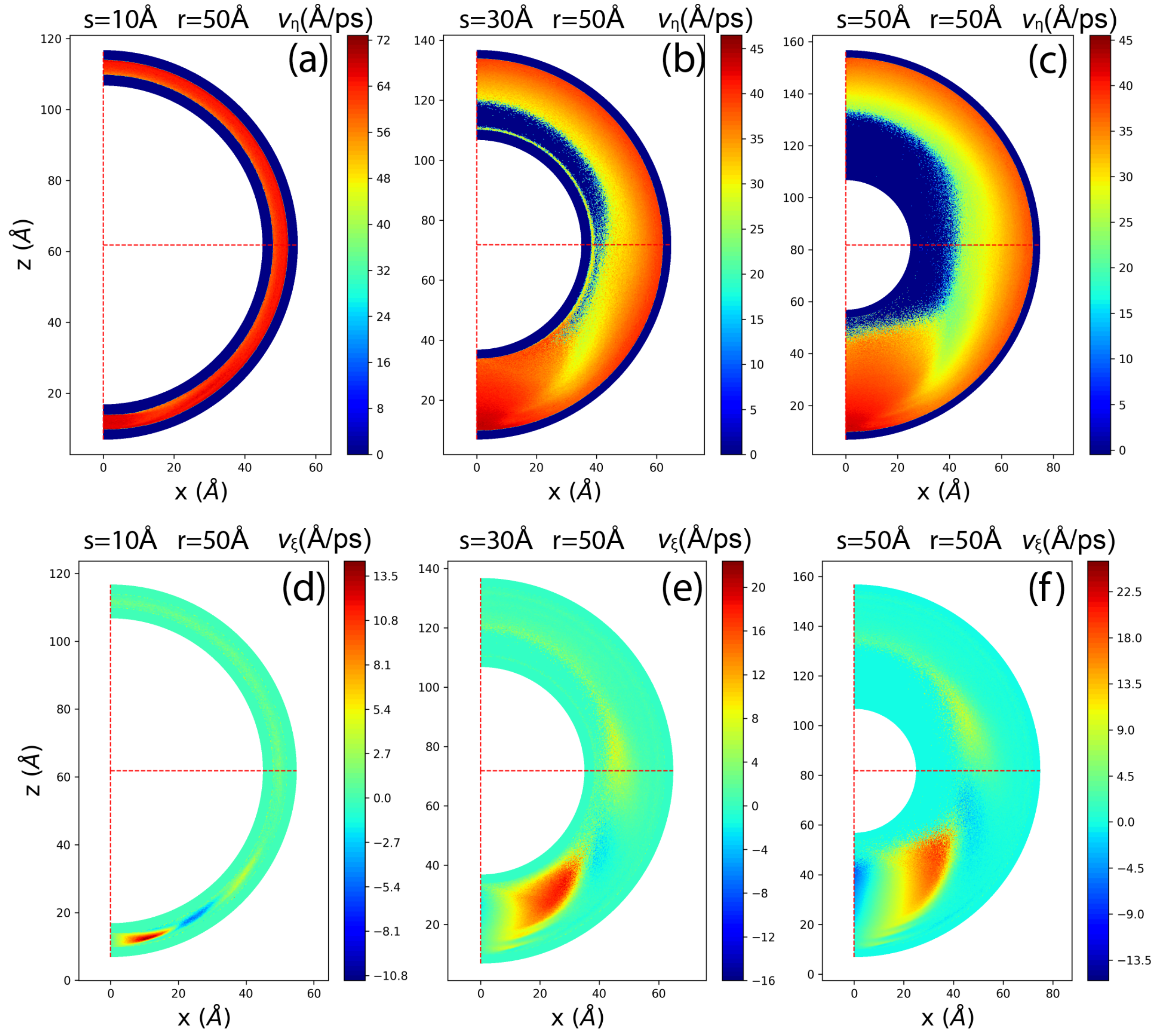

Figure S2: 2D flow velocity distribution in the nanochannel with $r = 50\text{Å}$ and different s. (a)(b)(c) are for the tangent flow velocities. (d)(e)(f) are for the normal velocities. Horizontal and vertical red dashed lines are the radial boundaries of region 1 and 3, note that straight region 2 disappears when $r = 50\text{Å}$.
